# Treatment of latent infection to achieve tuberculosis elimination in low-incidence countries

**DOI:** 10.1371/journal.pmed.1002824

**Published:** 2019-06-06

**Authors:** Jonathon R. Campbell, David Dowdy, Kevin Schwartzman

**Affiliations:** 1 McGill International TB Centre, McGill University, Montréal, Québec, Canada; 2 Respiratory Epidemiology and Clinical Research Unit, Montréal Chest Institute, McGill University Health Centre, Montréal, Québec, Canada; 3 Department of Epidemiology, Johns Hopkins Bloomberg School of Public Health, Baltimore, Maryland, United States of America

## Abstract

In a Perspective for the Tuberculosis Special Issue, Kevin Schwartzman and colleagues discuss the choices and implications for personal versus public health benefits when pursuing tuberculosis elimination in low-incidence countries.

**Summary points**

- Tuberculosis (TB) persists in the United States, Canada, and other high-income, low- incidence countries largely because of ongoing reactivation of latent TB infection (LTBI).
- TB elimination in low-incidence countries, defined as an annual incidence of ≤1 case per million, will require extensive screening and treatment of LTBI, including in people for whom the harms of LTBI treatment outweigh the likely benefits: for example, older foreign-born individuals with no recent travel/exposure. Ongoing migration from higher-incidence countries, as well as pockets of transmission in vulnerable subgroups such as prisoners, homeless persons, and drug users, will also continue to pose challenges for TB elimination.

- Policymakers in low-incidence countries face a choice between a utilitarian approach that tolerates individual net harm to advance public health goals and a patient-centered approach that values shared decision-making but will predictably result in failure to achieve TB elimination.
- While TB elimination is an important aspirational vision, the ethical implications of this goal—namely the implicit requirement to offer LTBI screening and treatment to individuals who are more likely to experience harm than good—merit careful reflection.

## Introduction

Tuberculosis (TB) remains an important public health problem in every country of the world. The World Health Organization (WHO) has developed the End TB strategy, which sets ambitious global goals: 95% decline in TB mortality and 90% reduction in TB incidence by 2035, compared to 2015 [1]. In high-incidence countries, reaching these goals will require much better access to timely diagnosis and treatment, as well as improvements in socioeconomic conditions and health systems. Low-incidence countries have already benefited from these improvements, reflected in marked decreases in TB-related morbidity and mortality over the last century: in Canada, incidence declined from a high of 103 per 100,000 in 1946 to less than 5 per 100,000 from 2000 onward. Similarly, TB-related mortality declined from 80 per 100,000 in 1924 to 0.4 per 100,000 by 2000 [2]. However, TB incidence has now leveled off in many low-incidence countries, and ongoing transmission now accounts for a minority of new cases [3,4]. This stagnation largely reflects the challenge of preventing ongoing reactivation of latent TB infection (LTBI), which causes most active TB in low-incidence countries [4,5].

**Fig 1:**
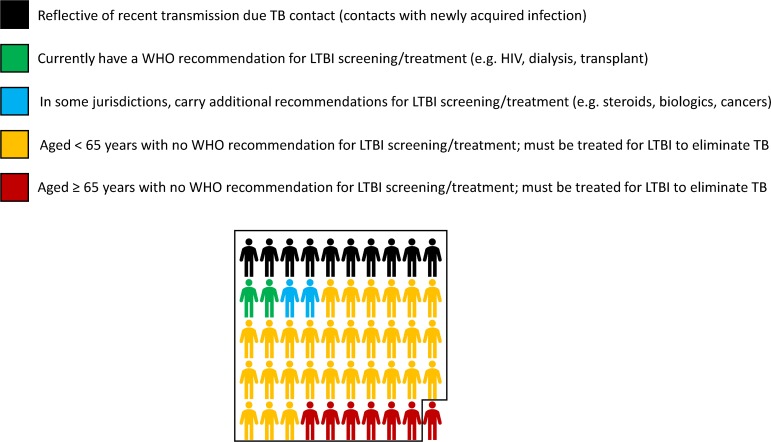
What would it take to eliminate TB in Canada? A pictorial description. Shown is the distribution of TB disease among risk groups. In Canada and other low-incidence countries, 70% of TB disease arises in foreign-born persons. Thus, 7 out of every 10 people in this figure would be foreign-born; in some countries, these individuals may also carry national recommendations for LTBI screening and treatment [10]. The box represents the 49 TB cases (out of every 50 now reported) that must be prevented in order to reach the elimination target of 1 case per million population [4,8]. HIV, human immunodeficiency virus; LTBI, latent TB infection; TB, tuberculosis; WHO, World Health Organization.

TB elimination is defined as an annual incidence of ≤1 case per million population [5]. For a low-incidence country like Canada, with 50 cases per million annually [6], eliminating TB will mean preventing 49 out of every 50 TB cases currently encountered. In Canada, much like many other low-incidence countries, roughly 70% of all TB occurs in foreign-born residents [7]—approximately 85% of which reflects reactivation of LTBI acquired abroad [4]. In Canadian-born persons, at least two-thirds of TB also results from reactivation [3]. Hence, only 20% of incident active TB in Canada results from an infection acquired recently within Canadian borders. Since the 1970s, Bacille Calmette–Guérin (BCG) vaccination has been provided only in settings where there is ongoing transmission, such as certain Indigenous communities. Persons who are currently prioritized for LTBI screening represent a small minority of the remaining 80% [8]. As the age distribution of people with LTBI evolves [9], an increasing proportion will be at high risk of adverse drug reactions (**Fig 1**).

Multiple modeling studies [11–13] likewise consistently demonstrate that, while halting transmission and treating active TB remain essential, elimination will require broad-based LTBI screening, and treatment will be needed on a scale never before undertaken. If this occurs, many situations will arise in which the anticipated risks of treatment outweigh expected benefits for the specific individuals concerned [14]. In evaluating whether to singularly focus on a goal of reducing TB incidence to below 1 per million, it is useful to explicitly consider the balance of risks to individuals and benefits to society that such a target would impose.

## Policy approaches to LTBI screening and treatment for TB elimination

The current WHO framework for TB elimination in low-incidence countries advocates an individual-level approach to LTBI screening and treatment [5]. This is motivated by the principle that an intention to screen an individual for LTBI is an intention to treat if positive, so that risks of treatment should be outweighed by anticipated benefits before screening is performed. Relying on robust epidemiologic evidence, WHO recommends LTBI screening when there is high certainty of substantial benefit (for example, close contacts of persons with active TB; human immunodeficiency virus [HIV]-infected persons). In low-incidence countries, this population of individuals is small. In Canada, for example, individuals recommended for screening by WHO criteria represent 2 of every 49 TB cases that must be prevented to eliminate TB. For the most part, these persons are already routinely screened and treated as part of standard clinical care [2,8,15,16].

WHO further suggests that screening be considered for other population groups in which the risk–benefit balance may be favorable in certain circumstances, such as prisoners, the homeless, and migrants from high-TB–incidence countries. It remains unclear whether the balance of risks and benefits consistently favors treatment for all such persons—but such people will need to be screened on a massive scale if TB elimination is to be achieved.

Although only conditionally recommended by WHO, national healthcare agencies in low-incidence countries like Canada [2], the United States [15], and the United Kingdom [16] recommend treatment in many groups in which there may be more limited benefit for the individuals concerned. These recommendations are influenced by modeling studies, which demonstrate population-level benefit when screening and treatment are applied systematically (e.g., migrants, persons with diabetes)—often predicated on a high assumed level of adherence [17,18]. This more “public-health–oriented” approach recognizes that elimination goals will not be achieved without expanding LTBI screening and treatment beyond those individuals who stand to gain the most.

The public-health–oriented approach is not without merit. For example, in countries like the United States and Canada, it has been widely applied to eliminate infectious diseases via childhood vaccination. Before vaccine introduction, 1 in every 50 people in the United States developed measles each year. This resulted in approximately 1 in 4 affected persons being hospitalized and 1 in 1,000 dying. Today, measles infects only 1 in every 1.5 million Americans each year [19]. Yet, the risks associated with vaccination, such as anaphylaxis (1 per million) [20], are still clearly outweighed by the immense public health benefit.

LTBI treatment is not measles vaccination, however. First, unlike measles vaccination, LTBI treatment does not induce an immune response and therefore cannot achieve herd immunity. Second, whereas the measles vaccine is almost universally immunogenic as delivered in low-incidence countries, LTBI diagnostics lack specificity and have a positive predictive value of <10% for future development of active TB. Thus, LTBI treatment will necessarily be given to many individuals, particularly in low-incidence countries, who do not stand to benefit, whereas nearly every individual appropriately vaccinated against measles receives immunity. Third, the risk of adverse events with LTBI treatment is an order of magnitude greater than for routinely administered vaccines [21]. Whether to favor the individual or the public health approach to TB prevention therefore requires further reflection, making the implications of each approach explicit.

## Benefits and risks of LTBI treatment

The primary benefit of LTBI treatment is reduced risk of TB disease with related morbidity and mortality, including both acute and longer-term complications such as chronic respiratory impairment [22]. Importantly, these benefits often accrue well into the future and are generally not detectable by persons with LTBI, their families, or their communities. Patient and community valuation of these potential benefits is critical to sustaining political and financial support for LTBI diagnosis and treatment programs. In formal terms, this valuation incorporates patient time preference, often described as a discount rate. For example, the commonly used discount rate of 3% [23] implies that preventing a TB case 10 years from now is valued 26% less than preventing a TB case tomorrow. Importantly, the discount rate varies considerably between individuals [24]. This means that faced with identical short-term risks and long-term benefits, some persons may choose LTBI treatment and others may decide against it, based on varying time preference. More generally, after understanding their personal risks and benefits associated with LTBI treatment, some individuals with LTBI will reasonably make an informed decision against treatment, based on rational preferences.

Another key benefit of TB prevention is reduced onward transmission. This is an important reason for offering LTBI screening and treatment to people who are homeless [25] and individuals in nursing homes [26]. Persons with LTBI may choose treatment because of benefits not only to themselves but to their family members, friends, and the broader community. In low-incidence settings, however, only a small minority of TB cases reflect ongoing transmission within country borders [27]; as a result, the added population-level benefit of reducing transmission, outside known outbreaks and congregate settings, is likely small.

Weighed against these benefits, the principal risk associated with LTBI treatment is drug-related adverse events, which range from bothersome to fatal. Unlike the benefits of LTBI treatment, adverse events occur during treatment, are immediately perceptible to patients and/or their providers, and may have lasting effects. The risk of significant adverse events during LTBI treatment is approximately 1 in 30 [21]; although much less frequent, hepatotoxicity can lead to liver transplantation or even death [28]. Foreign-born individuals will require treatment if TB elimination is to be attained, but many are over 65 years old; the risks of hepatotoxicity in these older individuals may be increased 3- to 5-fold [29]. Because these risks almost certainly outweigh the expected individual-level benefits, testing and treating such persons for LTBI implies an expectation that they put themselves at individual risk for the greater public health good.

Not considered outright adverse events, but still weighed by patients and providers in risk–benefit decisions, are the inconveniences associated with LTBI treatment. Risk–benefit models generally ignore patient inconvenience, yet convenience has an important (and understandable) impact on patient decisions [30]. For example, most treatment regimens are associated with recommendations to avoid alcohol intake [31]. A recent analysis of LTBI screening and treatment in foreign-born individuals with diabetes estimates that a 57-year–old individual would gain 0.0009 quality-adjusted life years (QALYs), equivalent to 8 quality-adjusted hours, if screened and treated for LTBI [32]. Many individuals would reasonably decide against three months of medication and alcohol abstinence to obtain this small benefit. Similar calculations could be made for the cost of attending clinic to take medications; for example, the societal cost of lost wages, healthcare worker time, and transport to clinic would likely not be considered cost-effective for a gain of 8 quality-adjusted hours in the example above.

## Individual risk versus group benefit: A case study

**Fig 2:**
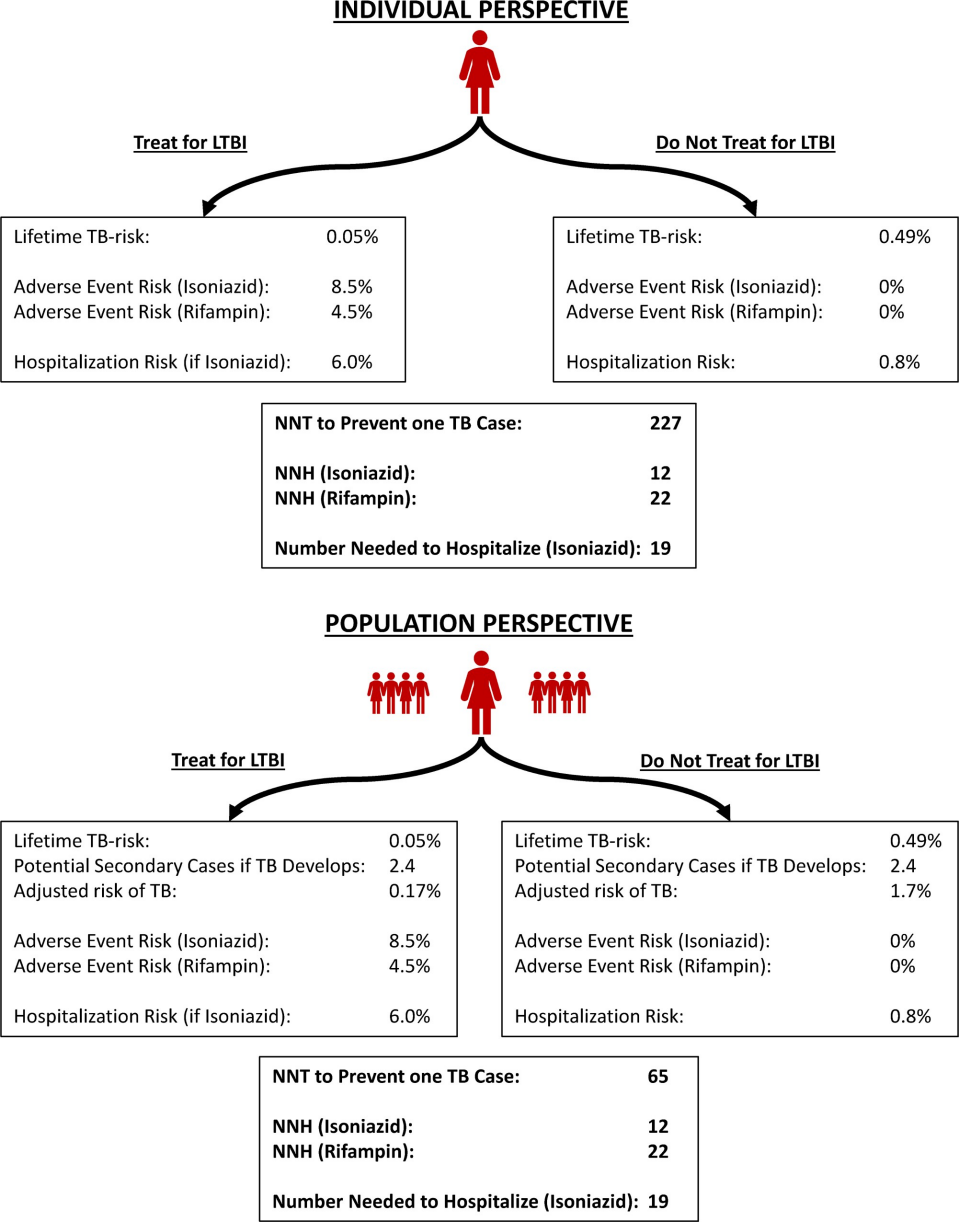
Risk–Benefit Considerations for Treatment of LTBI. Shown are the expected outcomes of treatment for LTBI versus no LTBI treatment. The individual is a 75-year–old woman with a 5-year life expectancy and a positive interferon-gamma release assay who immigrated to Canada from the Philippines 15 years ago and now resides in a 100-person nursing home. Two different perspectives are taken: an individual perspective and a population perspective [21,33,36,37]. LTBI, latent TB infection; NNH, number needed to harm; NNT, number needed to treat; TB, tuberculosis.

Providing LTBI treatment to individuals ≥65 years of age is a clinical challenge since they carry both the highest age-related risk of adverse events and highest age-related risk of TB [33,34]. In both Canada and the United States, this age group accounts for approximately one-quarter of TB cases [6,35]. If we are to reach the aspirational goal of TB elimination, persons in this age group must be included in screening and treatment initiatives [8]. A brief case study (**Fig 2**) examines two perspectives on this decision, demonstrating the risk–benefit considerations clinicians and their patients will increasingly face if we maintain the target of TB elimination.

When considering the risks and benefits of LTBI treatment from the individual perspective, the number needed to treat (NNT) to avert a case of TB substantially exceeds the number needed to harm (NNH) in terms of adverse event and hospitalization risk. This holds true even if a safer rifamycin-based regimen is used. However, from the population perspective, considerations may change. In this context, the development of active TB has substantial implications for those around the patient. The NNT to prevent one case of TB becomes considerably smaller, despite individual benefit being potentially outweighed by risks. As we pursue TB elimination, this type of choice will arise more frequently, including in individuals who live independently rather than in congregate settings [38].

The NNH depends on individual-level characteristics associated with treatment toxicity, regardless of whether the individual or population perspective is taken. However, the population-level NNT will also reflect key social factors: the number of likely contacts of the person(s) potentially treated, the characteristics of the individuals concerned, and the nature and intensity of interactions between them. Similarly, in the nursing home setting, the cost of treatment and contact investigation for active TB is high. In a study from Alberta, Canada, this totaled nearly $30,000 Canadian per active TB case among nursing home patients, compared with $500 per patient for LTBI screening and treatment [39]. However, the authors judged screening all entrants to be poorly cost-effective because of low reactivation risks with longstanding LTBI and very high short-term mortality [39].

## Implications

We argue that approaching LTBI treatment from an individual perspective is the most patient-centered and equitable way to proceed. The uncertainty inherent to LTBI testing among persons at lower risk of infection and disease, and the limited net individual benefit to most patients even in the era of shortened rifamycin-based regimens, likely justifies a more risk-averse approach as opposed to the more utilitarian public health framework. Yet, taking this approach implies an acceptance that—absent a new intervention (for example, vaccine) with a substantially lower risk profile—TB elimination will not be achieved within any of our lifetimes, let alone by the WHO target date of 2050. Additional quantitative research could be helpful in developing a TB incidence goal that could be achieved without subjecting any individual to an unfavorable balance of risks and benefits.

To achieve elimination, a “public health” approach to LTBI treatment will be necessary. This approach, however, carries underappreciated ethical implications, several of which have been raised above and elsewhere [14,40]. A purely utilitarian approach entails treatment of populations where, on a group level, the benefits of treatment outweigh the risks—even if some individuals experience net harm. In some situations, such as that of childhood vaccination, policymakers and public health authorities have made the ethical judgment that the substantial societal gains justify very limited individual risks. In the case of TB, however, elimination targets have been widely embraced, apparently without the same careful ethical reflection.

TB elimination as a vision and goal has had extremely positive impacts in terms of raising awareness of TB as a public health emergency, increasing political commitment to this neglected disease of poverty, and ensuring that funding is appropriately increased. As such, it is critical to maintain an ambitious public health vision to end TB as a global health threat. In doing so, however, defining elimination as one incident case per million population implies placing individual patients at greater risk than their anticipated benefits of treatment. Focusing on more modest near-term targets (e.g., 50% reduction in incidence over 10 years) could maintain the same level of ambition without the same ethically problematic implications. Policymakers, advocates, public health practitioners, and clinicians must also appreciate that risks and benefits will differ among diverse individuals as well as between groups and, in many cases, the risks of LTBI testing and treatment will outweigh the benefits. In these cases, absent a strong ethical mandate from society, we must share decision-making with patients, families, and communities based on clear and open communication and be willing to forego recommendations for treatment based on an understanding that both risk–benefit calculations and individual preferences may appropriately vary.

## Abbreviations

BCG: Bacille Calmette–Guérin
HIV: human immunodeficiency virus
LTBI: latent tuberculosis infection
NNH: number needed to harm
NNT: number needed to treat
QALY: quality-adjusted life year
TB: tuberculosis
WHO: World Health Organization
